# Early detection of *Solanum lycopersicum* diseases from temporally-aggregated hyperspectral measurements using machine learning

**DOI:** 10.1038/s41598-023-34079-x

**Published:** 2023-05-11

**Authors:** Michał Tomaszewski, Jakub Nalepa, Ewa Moliszewska, Bogdan Ruszczak, Krzysztof Smykała

**Affiliations:** 1 Faculty of Electrical Engineering, Automatic Control and Informatics, Department of Computer Science, Opole University of Technology, Prószkowska 76 Street, 45-758 Opole, Poland; 2 QZ Solutions Sp. z o.o., Ozimska 72A Street, 45-310 Opole, Poland; 3 Faculty of Natural Sciences and Technology, Institute of Environmental Engineering and Biotechnology, University of Opole, Ks. B. Kominka 6a Street, 45-032 Opole, Poland; 4 Department of Algorithmics and Software, Silesian University of Technology, Akademicka 16, 44-100 Gliwice, Poland; 5 KP Labs, Konarskiego 18C, 44-100 Gliwice, Poland

**Keywords:** Computer science, Electrical and electronic engineering, Plant sciences, Optics and photonics

## Abstract

Some plant diseases can significantly reduce harvest, but their early detection in cultivation may prevent those consequential losses. Conventional methods of diagnosing plant diseases are based on visual observation of crops, but the symptoms of various diseases may be similar. It increases the difficulty of this task even for an experienced farmer and requires detailed examination based on invasive methods conducted in laboratory settings by qualified personnel. Therefore, modern agronomy requires the development of non-destructive crop diagnosis methods to accelerate the process of detecting plant infections with various pathogens. This research pathway is followed in this paper, and an approach for classifying selected *Solanum lycopersicum* diseases (anthracnose, bacterial speck, early blight, late blight and septoria leaf) from hyperspectral data captured on consecutive days post inoculation (DPI) is presented. The objective of that approach was to develop a technique for detecting infection in less than seven days after inoculation. The dataset used in this study included hyperspectral measurements of plants of two cultivars of *S. lycopersicum*: Benito and Polfast, which were infected with five different pathogens. Hyperspectral reflectance measurements were performed using a high-spectral-resolution field spectroradiometer (350–2500 nm range) and they were acquired for 63 days after inoculation, with particular emphasis put on the first 17 day-by-day measurements. Due to a significant data imbalance and low representation of measurements on some days, the collective datasets were elaborated by combining measurements from several days. The experimental results showed that machine learning techniques can offer accurate classification, and they indicated the practical utility of our approaches.

## Introduction

Early detection of disease outbreaks in cultivation is not a trivial task. At the same time, it is crucial due to the possibility of applying appropriate remedial measures immediately after the infection occurs. Some plant diseases can reduce yields by 80% under favorable conditions for developing pathogens^1,2^, thus it is important to respond appropriately and promptly to disease symptoms. Conventional methods of diagnosing plant diseases are based on observing their symptoms. Unfortunately, they may be similar for a range of conditions, which increases the difficulty of correct diagnosis even for experienced farmers and requires detailed examination based on destructive methods conducted in the laboratory by qualified personnel^3^. Therefore, modern agronomy requires the development of non-destructive crop diagnosis methods, in particular, to shorten the time of detecting plant infections with various pathogens.

In this work, this research gap is addressed and the exploitation of the spectroscopic systems is proposed as a precision farming tool for the rapid and early detection and differentiation of *Solanum lycopersicum* (tomato) diseases. The contributions are two-fold: (1) the development of a machine learning algorithm allowing to accelerate the process of detecting crop infections by appropriate disease recognition before the appearance of the symptoms visible to the naked eye, and (2) the analysis of the effectiveness of such classification engines targeting selected tomato diseases in the days post inoculation (DPI) based on the acquired real-life hyperspectral data. To address the practical agricultural issue concerned with the regular scouting time, the method enabling to detect the infection in less than 7 DPI is introduced. Since the captured hyperspectral data is imbalanced, and for some DPIs the number of hyperspectral samples is under-represented, the aggregation strategies are introduced to elaborate the collective (and more representative) datasets combining measurements from several days.

The paper is organized as follows. The “Crop diseases classification using hyperspectral measurements on the consecutive days post inoculation” section reviews the current state of the art on detecting crop diseases from hyperspectral measurements. In the “Methods” section, the dataset acquired in this study is discussed, together with the proposed data-driven approaches for classifying selected tomato diseases from such data. The experimental results are presented in detail in the “Results” section, and they are further discussed (together with the limitations of this study) in the “Discussion” section. The final section concludes the paper.

## Crop diseases classification using hyperspectral measurements on the consecutive days post inoculation

The analysis of the condition of plants from a time perspective (e.g., on a day-by-day basis) is fundamental due to the need for early-stage disease detection. The topic of early detection of plant disease has been extensively researched in the literature, due to its practical applications in the agricultural industry^4,5^. However, the measurements were performed sporadically in the majority of published studies, e.g., 2–8 measurement sessions throughout the growing season^6,7^. Some researchers approach the analysis by indicating only the disease severity (e.g., early or late stage of the pathogen development) without considering the time that has passed since the infection and bypassing the analysis of the disease development in terms of time^8^. There are works which point out that the measurements should be performed within a specific time interval since the infection (6–42 DPI), however, the reported results commonly omit the analysis for particular days post inoculation^9^.

There exist studies which were conducted with a high measurement frequency, as with the methods applied in the presented work. In the work by Gold et al.^10^, the measurements were taken every 12–24 h for 5–7 DPI. Xie et al.^5^ performed weekly measurements between 11 and 60 DPI, and the results were analyzed independently for the visible and near-infrared (VIS-NIR) and short-wave infrared (SWIR) wavelength ranges. For the VIS-NIR range, the best $F_1$ score of the binary classifier (Support Vector Machine, SVM) distinguishing the diseased and healthy plants was 0.79 on 32 DPI and 39 DPI, whereas for the SWIR range, it amounted to 0.83 for 60 DPI. Also, in the case of^11^, weekly hyperspectral measurements of the VIS-NIR were performed between 6 and 42 DPI. However, the analysis from a time perspective was limited to indicating the time of visible disease symptoms appearance. An exciting approach was suggested in the work of Peng et al.^12^, where the binary disease classification on multispectral data for two varieties of Cassava was applied at 4-time points after inoculation. The results varied depending on the variety, applied classification methods and the time point of interest, and ranged from 50 to 92% and 50–85% accuracy for the TME204 and the Kiroba varieties, respectively, for 28 DPI.

The unprecedented success of deep learning techniques is observed in an array of scientific and industrial fields due to their intrinsic representation learning capabilities, with precision farming not being an exception here. In the experiments of Nguyen et al.^13^, 40 hyperspectral images were taken at 4-time points between the 7th and 8th of October in 2019. Firstly, the images were processed using two-dimensional and three-dimensional convolutional neural networks (2D-CNN and 3D-CNN) to extract deep features. Secondly, SVM and Random Forest classifiers were trained over such automatically elaborated feature vectors, with the latter offering higher-quality classification. However, it should be noted that due to the small amount of measurement data, the results were unacceptably low. The accuracies for the training dataset were 71% and 75% for 2D-CNN and 3D-CNN, respectively, and the best accuracy for the test dataset reached 50%. Also, in the Cen et al.^14^ research, the measurements in 4-time points were conducted (on May 7th and 20th, June 6th and 19th in 2021). Four feature extraction methods were examined in the study: Principal Component Analysis, Sequential Forward Selection, Simulated Annealing and Genetic Algorithms (GAs), whereas an SVM was used as the primary classifier. The combination of Genetic Algorithms and Support Vector Machines (GA-SVM) elaborated the best accuracy of 91% and 93% for leaves and stems, respectively. The study was re-performed in 2022, and the same approach achieved 89% and 80% accuracy for leaves and stems, respectively. The combination of GAs and SVMs was also exploited in the research of Nagasubramanian et al.^15^ as a model suitable to tackle the abovementioned issue of plant disease classification. The measurements were conducted 5 times in the period of 15 days after infection (3, 6, 9, 12 and 15 DPI). The obtained $F_1$ score (over the test dataset) was equal to 0.97—the study concerned the classification of infected stems with visible disease symptoms. In Ref.^4^, the authors investigated the measurements within the first 5 DPI. The following binary classifiers were used: K-Nearest Neighbors, C5.0 (an algorithm used to generate a Decision Tree) and FR-KNN (Feature Ranking K-Nearest Neighbors). The best classifier (FR-KNN) obtained the classification accuracy between 94.44 and 97.22%. However, the results for the individual measurement days ranged from 10 to 58.33%.

Over time, research on crop disease changes using hyperspectral data was also carried out on a microscopic scale^16^. In Ref.^16^, daily measurements were made from 3 to 14 DPI using a measuring device consisting of a hyperspectral camera and a microscope. The Simplex Volume Maximization (SiVM) was used. This research focused on analyzing the change in the spectrum of infected plants depending on the time elapsed after inoculation which is an important research gap that has been commonly ignored in other studies, but it is of utmost practical importance to accelerate the process of detecting infections by appropriate disease recognition.

## Methods

The dataset used in our study consists of hyperspectral plant measurements of 2 varieties of *Solanum lycopersicum*: Benito (Bejo Zaden, highly resistant to Late Blight) and Polfast (Bejo Zaden, tolerant to many diseases). Such a selection of varieties was proposed to allow for very early disease detection and thus an early decision on protection in the case of plants with disease resistance or tolerance. Including these varieties in the same dataset made it possible to obtain non-specific symptoms noticeable on plants for a long time. We consider this type of symptoms to be not detectable in the field with the help of classical visual assessment. The plants were inoculated by five various pathogens (Table 1): *Colletotrichum coccodes* (causes Anthracnose, *AN*), *Alternaria solani* (Early Blight, *EB*), *Pseudomonas syringe pv. tomato* (Bacterial Speck, *BS*), *Phytophthora infestans* (Late Blight, *LB*), *Septoria lycopersici* (Septoria Leaf Spot, *SL*).

**Table 1:** Summary of typical sympthoms of analyzed *S. lycopersicum* diseases.

Acronym	Disease	Pathogen	Visual symptoms
*AN*	Anthracnose	*Colletotrichum coccodes*	
*EB*	Early Blight	*Alternaria solani*	
*BS*	Bacterial Speck	*Pseudomonas syringe pv. tomato*	
*LB*	Late Blight	*Phytophthora infestans*	
*SL*	Septoria Leaf Spot	*Septoria lycopersici*	

The control plants which were not infected with any pathogen constituted an additional group. A total of 36 plants were investigated, with six plants in each of the abovementioned groups. Plants were cultivated in a fully controlled environment. Seeds were obtained from Bejo Zaden. Plants were planted in separate pots two weeks after seeding. Plants were inoculated 2–3 weeks after planting. The whole process was repeated in three separate rounds in phytotrons (a controlled environment). The plants were cultivated at 18–20 $^{\circ }$C, 60–80% humidity, and a photoperiod of 16 h with 200 $\upmu$mol m$^{-2}$ s$^{-1}$. All acquired spectral measurements were conducted in the same, controlled environment.

The pathogens were investigated on leaves and fruits to appropriately measure them. Each pathogen determination included its microscopic analysis, pathogen isolation, and species determination. The diseased parts were extracted from leaves or fruit, and they were surface-disinfected for 3 min in 2% sodium hypochlorite. The sample was dried, cut into four smaller fragments and placed on a PDA medium. After 7–10 days, the grown cultures were assessed and assigned to the offenders’ species. All plant experiments have been conducted at the Department of Vegetable Crops at Poznań University of Life Sciences following the European Standards for plant investigation. Plant material have been obtained, and all experimental protocols in the present study comply with international, national, and institutional guidelines.

### Inoculation methods

For each species of pathogen, spores or bacteria of that species were isolated and cultured on at least six individual plates, not less than 9 cm in diameter. All pathogens were cultured in a standard growth media under sterile conditions. The cultivation and storage of pathogens proceeded under controlled conditions: the constant temperature at 20 ± 1 $^{\circ }$C and constant air humidity above 70%. An inoculum for infecting each pathogen species was prepared by mixing the growth medium and mycelial spores or bacteria. The suspension was diluted to a concentration of $10^{5}$ to $10^{6}$ spores/cfu (colony-forming unit) per 1 ml and applied to plants by immersing the plants in the solution. The pathogens came from the Pathogen Bank (Institute of Plant Protection—National Research Institute, ul. Władysława Węgorka 20, Poznań, Poland) or were isolated from sympthomatic tomato fruits. The pathogen bank included isolates *Colletotrichum coccodes* (CBS 103.16), the culprit of anthracnose, *Septoria lycopersici* (CBS 354.49), the culprit of septoria, *Phytophthora infestans* (CBS 429.90), the culprit of late blight and *Alternaria solani* (CBS 142772), the culprit of early blight, where CBS number refers to the Centraalbureau voor Schimmelcultures strains database. *Pseudomonas syringae pv. tomato*, the culprit of bacterial speck, was isolated from tomato fruits (originating from Kościelec, Poland).

### Research crops and the measurement experiment

**Figure 1:**
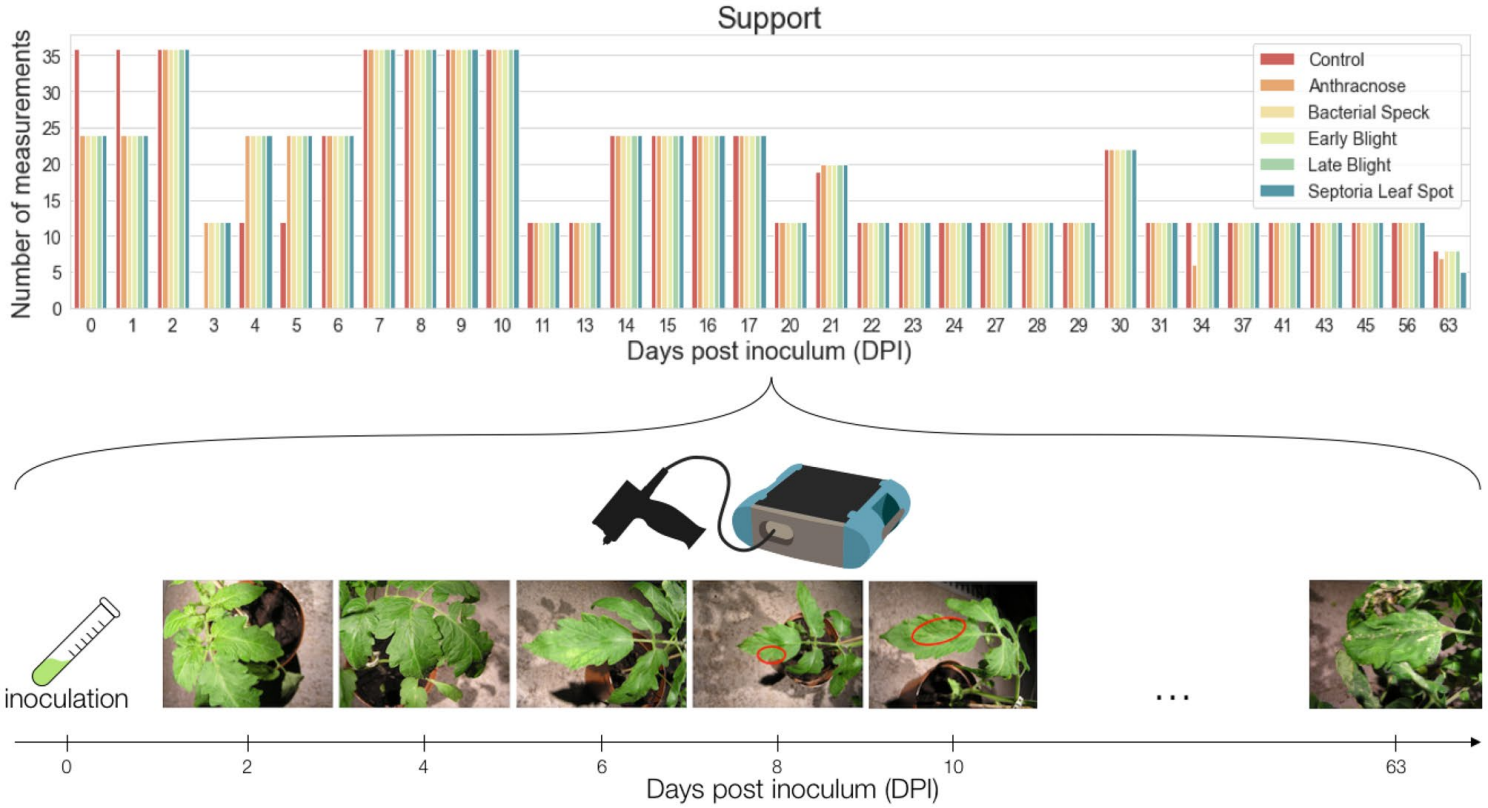
A high-level scheme of the measurement experiment.

The possibility of diagnosing the diseases on a single measurement, without considering the time perspective analysis, was evaluated in a similar study utilizing the hyperspectral data from a semi-controlled environment^17,18^. Here, we focus on the time perspective approach to the hyperspectral dataset which was obtained in the controlled environment during 34 measurement days between inoculation and 63 DPI (Fig. 1).

The measurements were taken almost every day after inoculation (DPI = 0, 1,..., 63), with particular emphasis put on the first 17 days (day-by-day measurements). The initial data preprocessing consisted in isolating incorrect data, such as incorrectly calibrated measurements, and those that were not the subject of the study (e.g., environmental background) from the correctly performed measurements. Then, the metadata encoded in the measurement reference number was extracted and added to the dataset^19^.

Selected leaves from each plant were scanned 5 times by a spectroradiometer in a single measurement series. In total, disregarding device write errors, 72156 records were obtained in the measurements series. As a result of the preprocessing operations, the number of primary measurements was reduced from **72156** to **58186** by screening out the measurement errors, then to **11634** by determining the medians of the measurement series. Finally, the measurements made from the height of 5 cm were selected as the least noisy (i.e., affected by the background signal) and, at the same time, the most reliable, which resulted in obtaining a dataset of **3877** unique, reliable, and fully described measurements.

### Reflectance measurements

The hyperspectral reflectance measurements were performed using a high-spectral-resolution field spectroradiometer ASD FieldSpec 4 Hi-Res (Malvern Panalytical Ltd., Malvern, UK) operating in the spectral range of 350–2500 nm. The spectral resolution of the device was 3 nm at 700 nm, 8 nm at 1400 nm, and 8 nm at 2100 nm. The spectroradiometer was calibrated before every measurement session using a white reference panel (spectralon) and dark current. The white reference panel reflectance was equal to at least 99% in the 400–1500 nm range and at least 95% in the range 250–2500 nm^20^. The device was powered up at least 30 min before every calibration and measurement session. The measurements were obtained from the 5, 30 and 60 cm heights with an artificial halogen light source. The field of view of the optical fibre was 25$^\circ$. Each series of measurements was documented photographically and visually assessed.

### Dataset structure

**Figure 2:**
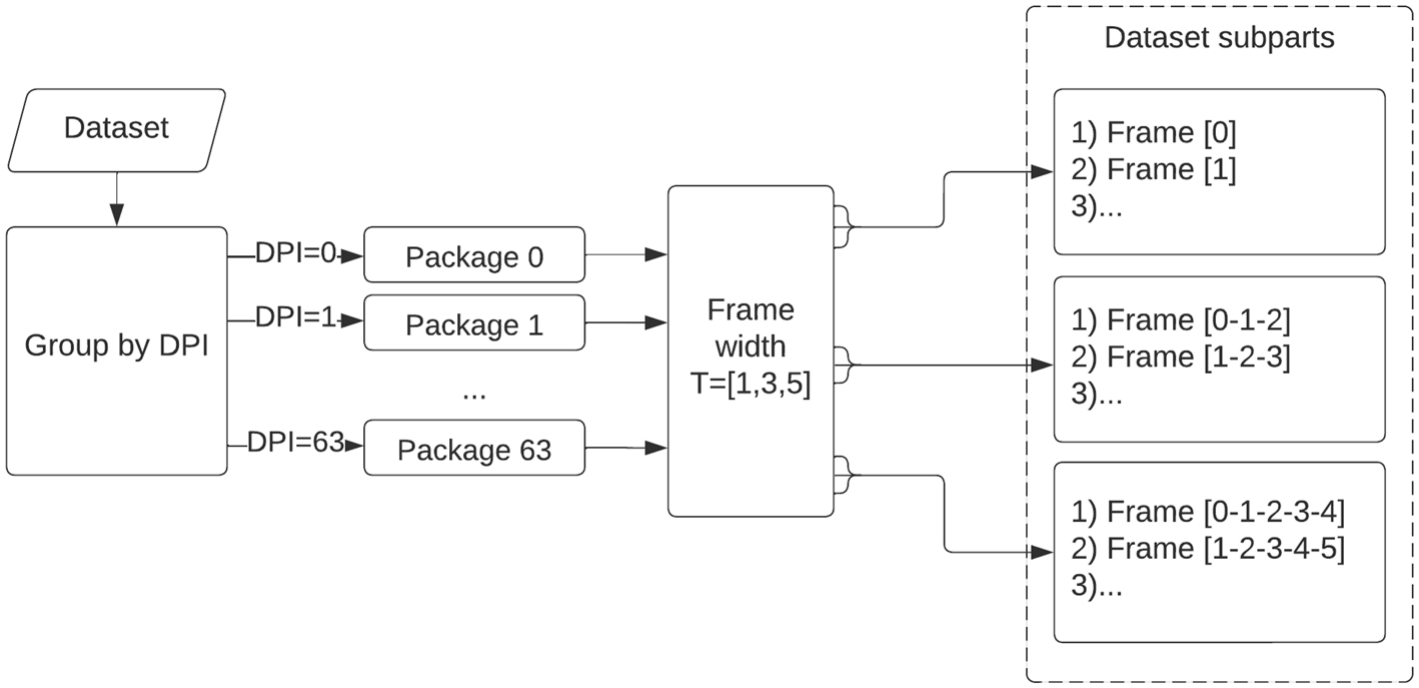
The process of creating data subsets for the analysis of time intervals (DPI unfolds to the days post inoculation).

While creating the dataset, a systematic data collection was hindered, among others, due to the measurement errors and device calibration errors. Therefore, despite exercising every care during the procedure, the measurement samples were obtained in different numbers on consecutive days post-inoculation. Due to the low representation of measurements on some days (Fig. 1), e.g., days 3, 11, 13, 20, the measurements from several days were aggregated into the joint datasets. Depending on DPI (0, 1, 2, ..., 63), the dataset was divided into the measurement packages, according to the diagram shown in Fig. 2. The measurement package is a collection of the measurements from a given day containing all spectral ranges for all plants from the observed phytotrons. Then, the data frame width was determined ($T = [1, 3, 5]$), being the interval covering the specified number of consecutive measurement days.

The frame widths were designed to detect the disease as early as possible, i.e., at the stage when the first symptoms of the disease appear or are not yet visible to the bare eye^21^. In practice, this means less than 7 days. After grouping the dataset by *DPI* and determining the size of *T*, the dataset was split into subparts, as presented in Fig. 2. Due to the lack of measurements, it was not possible to conduct the classification for every *DPI*—for example, for $DPI=3$, no control measurements were recorded due to the incorrect calibration. In the training process, the control measurements were exploited as well. Finally, the total number of samples was 7384, 21016 and 33016 for $T=1$, $T=3$ and $T=5$ (Table 2).

**Table 2:** Total number of samples for every experimental variant investigated in the study. The training/test dataset split is reported as well.

	T = 1		T = 3		T = 5	
	Train	Test	Train	Test	Train	Test
*CONTROL* vs. *ALL*	854	420	2378	1172	3694	1820
*CONTROL* vs. *AN*	812	400	2320	1144	3652	1800
*CONTROL* vs. *BS*	822	404	2346	1156	3694	1820
*CONTROL* vs. *EB*	822	404	2346	1156	3694	1820
*CONTROL* vs. *LB*	822	404	2346	1156	3694	1820
*CONTROL* vs. *SL*	818	402	2342	1154	3690	1818
Sum	4950	2434	14078	6938	22118	10898
Overall	7384		21016		33016	

Significant values are in [bold].

The experimental study targets six experimental classification variants:

1. *CONTROL* measurements vs. measurements of *ALL* diseased plants (*AN* : *SL*),
2. *CONTROL* vs *AN* (Anthracnose),
3. *CONTROL* vs *BS* (Bacterial speck),
4. *CONTROL* vs *EB* (Early blight),
5. *CONTROL* vs *LB* (Late blight),
6. *CONTROL* vs *SL* (Septoria leaf spot).

Variant 1 (*CONTROL* vs. *ALL*) represents a dataset composed of randomly selected measurements of the remaining datasets (*AN* : *SL*).

**Figure 3:**
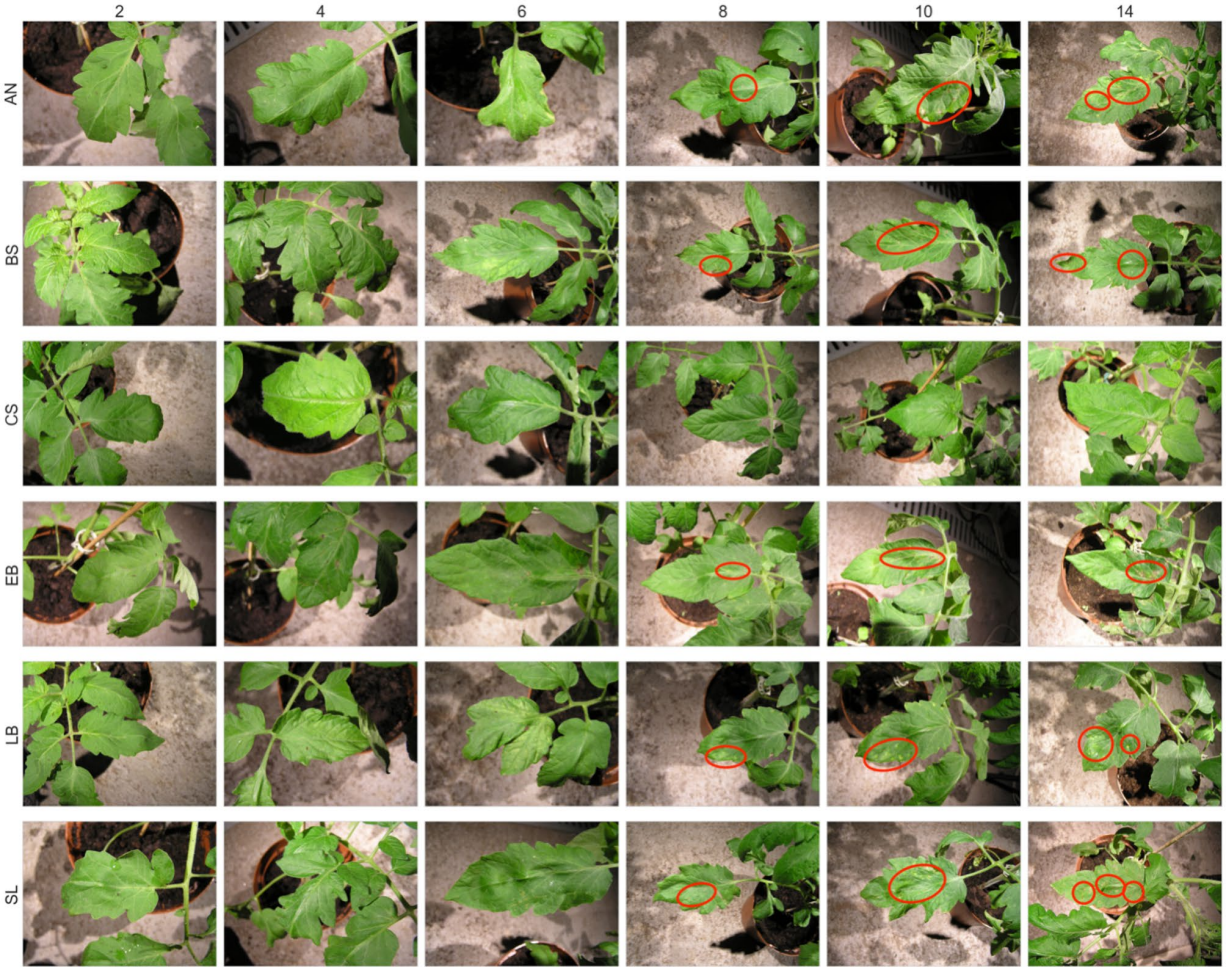
Comparison of reference photos of the tested plants during the development of diseases in the first two weeks after inoculation. Visible lesions are marked with a red outline; column numbering indicates the number of days since inoculation; the lines *AN*, *BS*, *CS*, *EB*, *LB* and *SL* indicate the disease cases.

The reference photos of the studied plants (Fig. 3) present the development of individual diseases in the time since inoculation. The small number of lesions visible to the naked eye in the first days after infection indicates the difficulty and significance of creating non-invasive systems for detecting diseases at an early stage of their development. It is a challenging task for a farmer to assess the condition of a given crop or to identify the type of pathogen based on visual assessment, but the correctness of that evaluation and performance of adequate treatments may significantly affect the future harvest^1,2^.

### Classification methods

In this work, the classic machine learning classifiers are exploited: Random Forests, Light Gradient Boosting Machines (LGBM), and Ridge classifiers. In random forests, an ensemble of independently trained decision trees are utilized to enhance the prediction abilities of the base learners—this technique was used to tackle an array of agricultural tasks^17,18,22^. In a similar vein, LGBMs utilize gradient descent to minimize the loss during adding models to an ensemble and work sequentially, where every weak learner (e.g., a decision tree) learns from the predecessor’s mistake. LGBMs were used to create various models in agriculture, among others, in Refs.^23,24^. Finally, the Ridge classifier is a linear classification algorithm based on Ridge regression^25^. Here, the binary classification in each of the analyzed variants is targeted. The input feature vectors for the classifiers were the reflectance values in the spectral range between 350 and 2500 nm (2151 spectral features) and the output was binary information about the plant condition (healthy or diseased). An important aspect of this study was to build a well-generalizing machine learning model while taking into account a large set of predictors. Here, the process of the hyperparameters’ tuning of the models was performed following the random search approach—as an example, for the Random Forest classifier, a range of values from 200 to 2000 for the number of trees (estimators) was random-searched, and the best result for each aggregation method was obtained for 1400 trees (T = 1), 1800 trees (T = 3), and 400 trees (T = 5).

To quantify the generalization capabilities of the machine learning models, the $F_1$ score (Eq. 1), being the harmonic mean of precision (Eq. 3) and recall (Eq. 4), as well as accuracy (Eq. 2) were exploited:

1 $$\begin{aligned} F_1 score= & {} \frac{2\cdot TP}{2\cdot TP + FP + FN}, \end{aligned}$$

2 $$\begin{aligned} Accuracy= & {} \frac{TP + TN}{TP + FP + TN + FN}, \end{aligned}$$

3 $$\begin{aligned} Precision= & {} \frac{TP}{TP + FP}, \end{aligned}$$

4 $$\begin{aligned} Recall= & {} \frac{TP}{TP + FN}, \end{aligned}$$

where: *TP* is the number of true positives, *TN* is the true negatives, *FP* is the false positives, *FN* is the and false negatives.

The weighted-averaged $F_1$ score (Eq. 1) was calculated by taking the mean of all per-class $F_1$ scores while considering data imbalance. The other metrics: precision (Eq. 3), recall (Eq. 4) and accuracy (Eq. 2) have been calculated using the same set of modeling results.

## Results

The microbiological analyses confirmed infection by particular pathogens on the investigated tomato plants. Visual disease recognition was difficult due to the high *LB* resistance and tolerance to other diseases, and symptoms developed more slowly. However, the first symptoms were possible to be seen in the 6–8th *DPI* mostly as chlorotic stains, and more clearly they were visible in the 14th *DPI* (Fig. 3). Our experimental study is split into two separate phases. In the first phase, the capabilities of the machine learning classifiers were investigated. Furthermore, the above-mentioned method of aggregating data from several days was exploited to verify the classification performance of such techniques. The second phase focused on a detailed analysis of the results obtained from a time perspective based on *DPI* and on determining the moment of receiving reliable information on the infection according to the variants mentioned above. All classification results were analyzed from two perspectives:

- *CONTROL* vs all diseases (*ALL*), where $ALL = AN + BS + EB + LB + SL$,
- *CONTROL* vs respective diseases (partition of the dataset containing the measurements of infected plants): *AN*, *BS*, *EB*, *LB*, *SL* denoting respectively: *AN* vs *CONTROL*, *BS* vs *CONTROL*, *EB* vs *CONTROL*, *LB* vs *CONTROL*, *SL* vs *CONTROL*.

To evaluate the classifiers for different pathogens, the $F_1$ score was exploited for each trained model calculated over the test set. The $F_1$ scores were also utilized to highlight the “right” moment of the experiment when the classification can be considered trustworthy, giving a significant reason to raise a decision on when the plant intervention should be taken. Although the problem of selecting an appropriate threshold for the $F_1$ score in this context is not trivial, as there is no single “gold standard” here, there are general rules regarding the $F_1$ score values, according to which reaching the level of 0.8 proves the model to be sufficiently well-functioning^26^. Several studies reported similar $F_1$ score levels as satisfactory regarding the pathogen detection process. Xie et al.^5^ received the $F_1$ score of 0.79 while analyzing hyperspectral data from the VIS-NIR range, and $F_1$ score amounted to 0.83 when classifying pathogens over SWIR. Some other works^15^ resulted in even better classification performance ($F_1$ score above 0.9), but they obtained those results while investigating plants in the later period. Therefore, to reflect the current state of the art in the field, the $F_1$ score of 0.8 was considered to be the acceptance threshold in this study (presented as the orange dashed line in the resulting plots).

### Classification performance for different data aggregations

**Figure 4:**
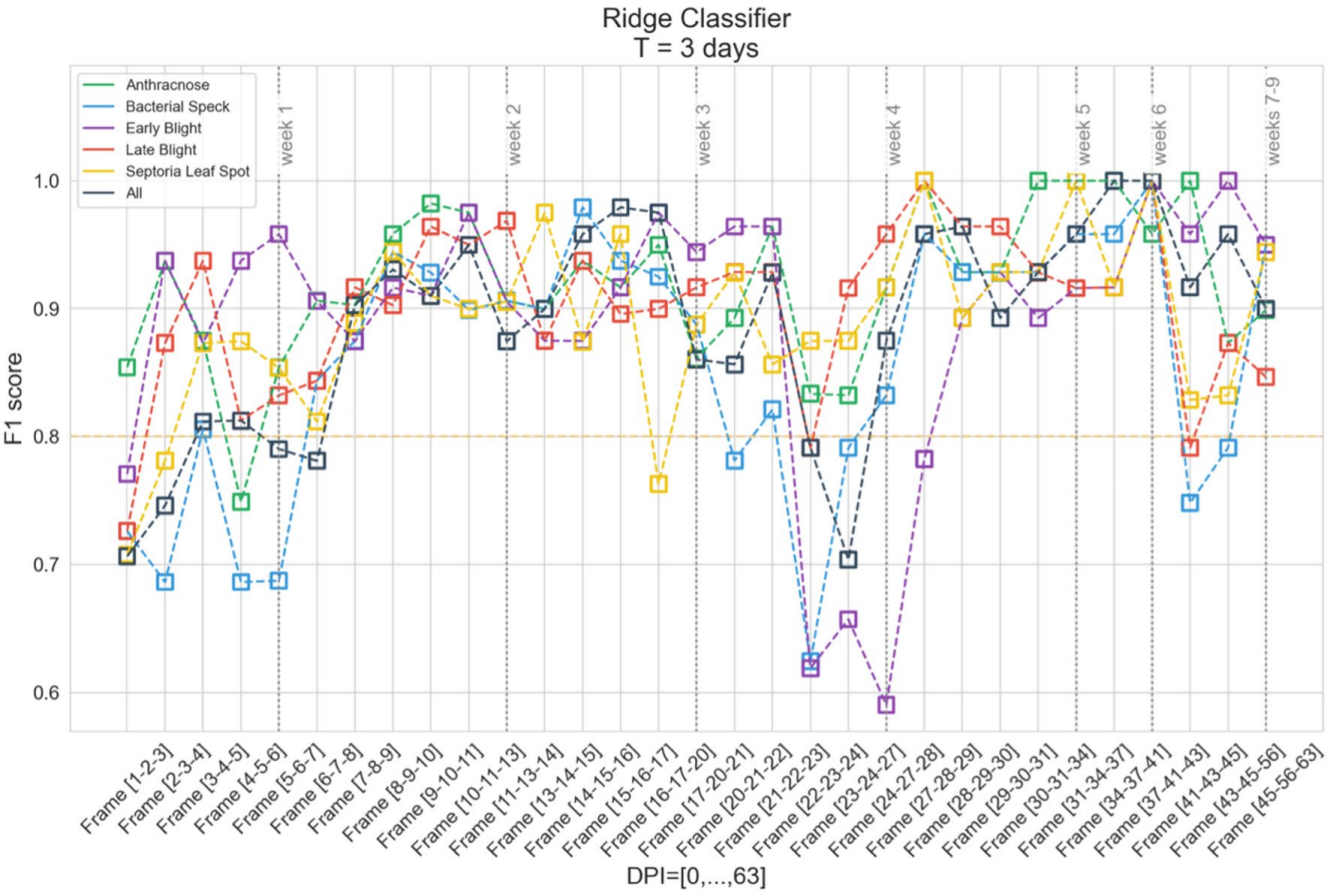
The results obtained for the Ridge classifier for individual frames and $T=3$.

In order to evaluate the effectiveness of the selected classifiers in time, the training process was performed for the data obtained on consecutive days past inoculation. These data were simultaneously aggregated according to the above-discussed method with $T=1, 3$ and 5 days, respectively. Figure 4 presents the example results obtained using the Rigde classifier and the aggregation of T = 3. The dataset was split into the training and test sets in the proportion of 2 : 1—all presented results were elaborated for the unseen test set.

**Figure 5:**
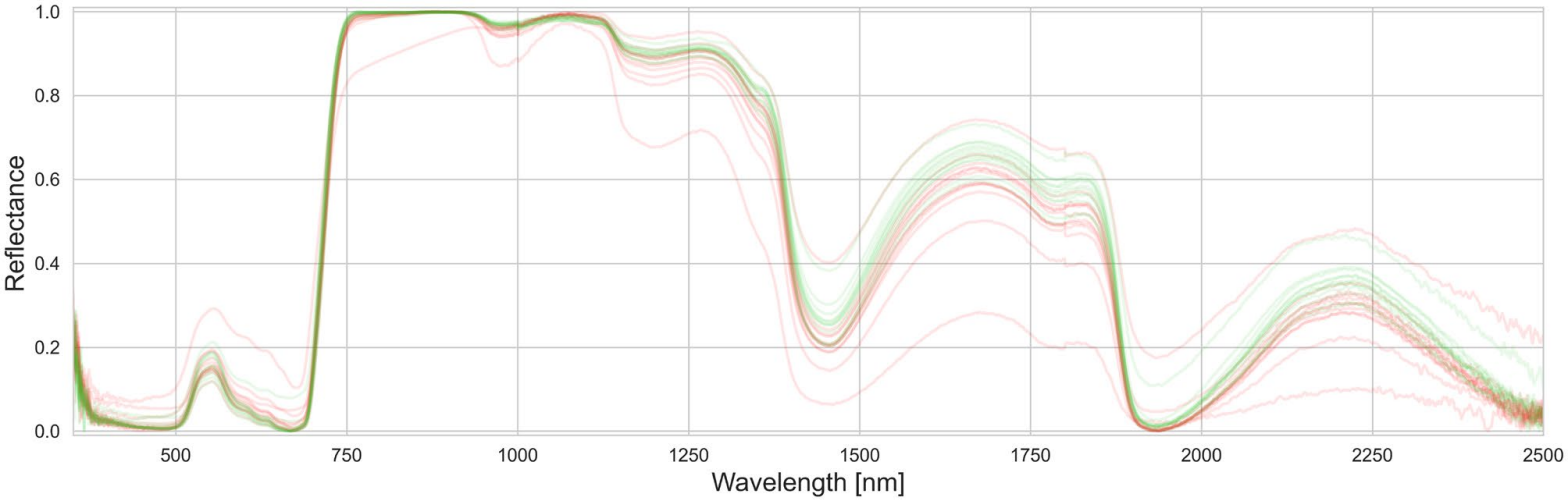
Early blight (red) and *CONTROL* (green) in DPI=23.

In this case, the results of the $F_1$ score were obtained in the range from 0.59 to 1.00. Its medians for respective variants were 0.9 for *ALL*, *BS*, and *SL*; 0.91 for *EB* and 0.92 for *AN* and *LB*, with a standard deviation between 0.069 and 0.101. There is a noticeable decrease in the results for *BS* and *EB* for frames [22-23-24] and [24-27-28], which was likely caused by an incorrect calibration error not detected at the preprocessing stage. An incorrectly performed measurement is visible in Fig. 5 as an outstanding record.

**Figure 6:**
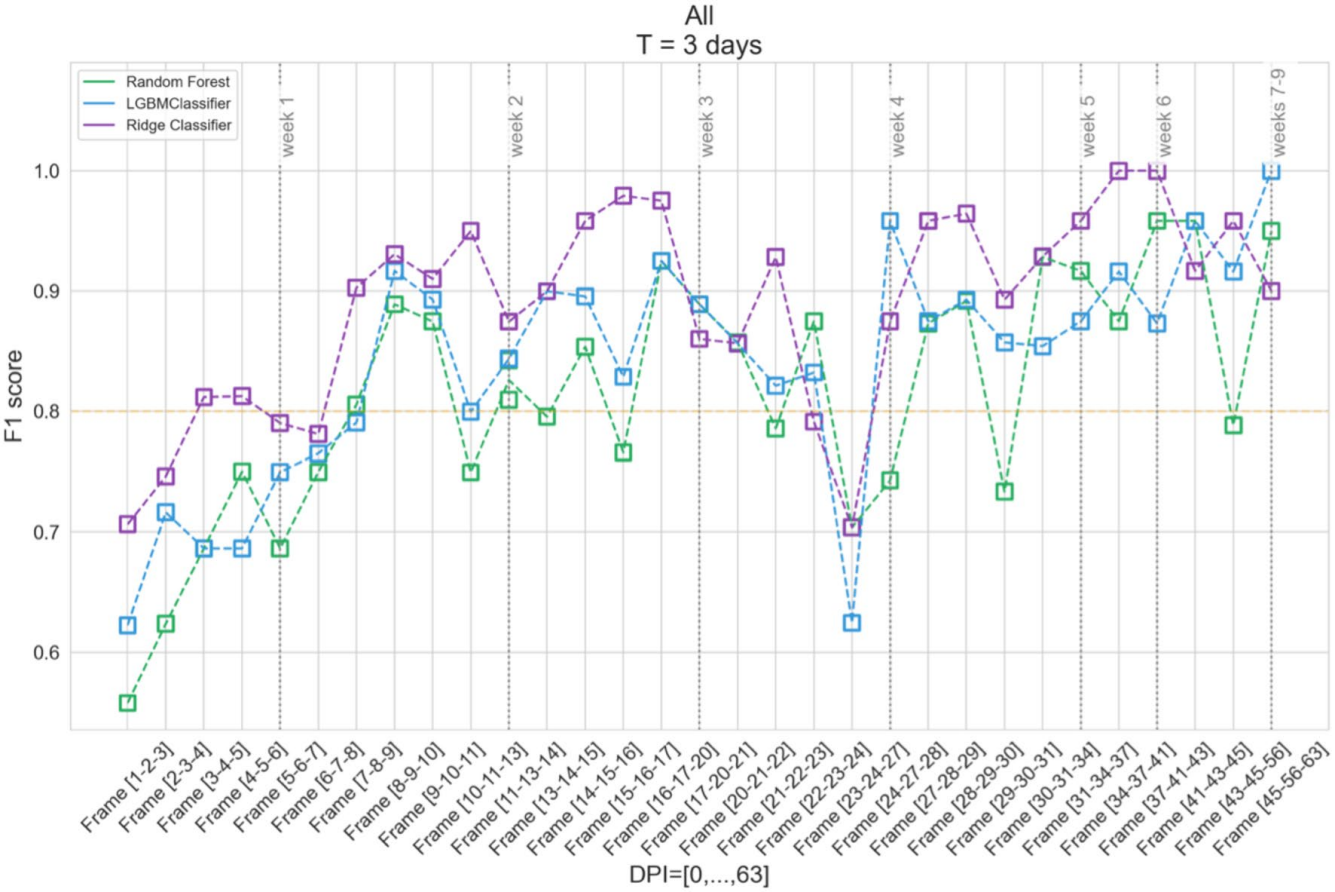
The results obtained using the investigated classifiers for the *ALL* vs *CONTROL* variant for individual frames and T = 3.

In Fig. 6, the aggregation interval T=3 was also assumed but showed the results for different classifiers for the *ALL* diseases vs *CONTROL* variant. Metrics described in the “Methods” section have been calculated for every trained classifier. As an example, a confusion matrix for the best Random Forest model for aggregation interval T = 3 (DPI = [10, 11, 13], LB) achieved: $F_1$ = 0.974, *Accuracy* = 0.975, *Precision* = 0.95, *Recall* = 1.0 calculated on the test set. The following values were obtained: TP = 47.5%, FP = 2.5%, FN = 0.0%, TN = 50.0%. These results can be considered promising in terms of machine learning models due to their good prediction of true positives (47.5%) and negatives (50.0%), with only a minor type I error (2.5%), and no type II error. Median scores for all classifiers are presented in Table 3. The significant increase in the $F_1$ score for the presented approach from 0.6 to 0.9 in the first 12 iterations (2 weeks after inoculation) is particularly noticeable. The median of the $F_1$ score for individual classifiers ranged from 0.83 (Random Forest) to 0.9 (Ridge classifier), with the standard deviation of 0.090–0.100. There was a significant improvement in the results over the single-day sampling approach. The similar results were obtained for the aggregation time of T = 5 days.

**Table 3:**
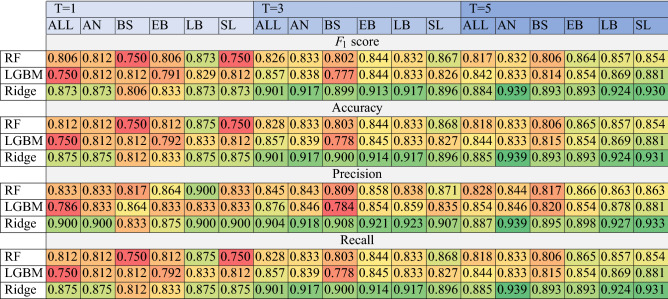
Median of the $F_1$ scores, accuracy, precision and recall for every classifier (Random Forest, LGBM, Ridge classifier) obtained for every disease variant (*ALL*, *AN*, *BS*, *EB*, *LB*, *SL*) and every data aggregation variant ($T=[1,3,5]$). The red color represents the lowest scores, whereas the green color—the highest.

The results obtained for all classifiers (Random Forest, LGBM, and Ridge classifier), and for the data aggregation periods ($T=1$, 3 and 5 days) are gathered in Table 3. Here, median values are presented in both experimental scenarios, i.e., all diseases and for each disease separately. The median $F_1$ scores range from 0.75 to 0.94—for the aggregation periods of $T=3$ and $T=5$, the obtained results were very consistent, with the Ridge classifier outperforming other models. Further analyses focused on the aggregation periods, but it should be noted that the aggregation period of $T=3$ provides a reliable diagnosis earlier and allows for making treatment decisions in a shorter time due to the less time needed to collect the necessary data (3 days). For completeness, the results of accuracy, precision and recall for an equivalent scenario as mentioned above were presented in the following rows in Table 3.

### Early detection of the pathogen infections

**Figure 7:**
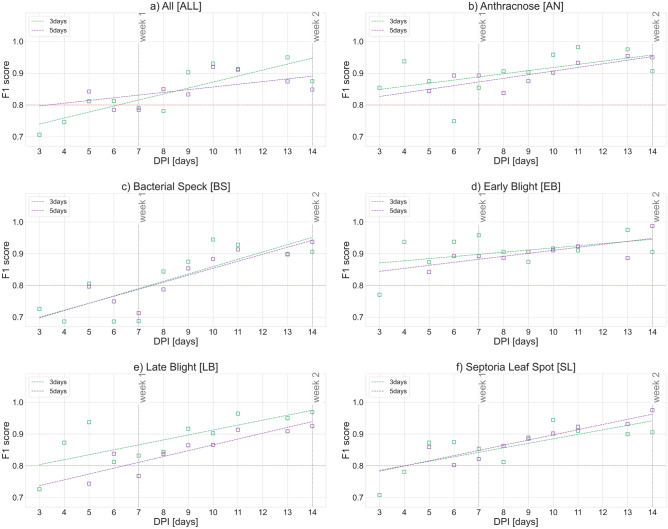
Increase of the classifier effectiveness in consecutive days from inoculation—variant *CONTROL* vs (**a**) *ALL*, (**b**) *AN*, **c** *BS*, **d** *EB*, (**e**) *LB*, (**f**) *SL* (linear model—dashed line); green and purple colors represent results for aggregation periods $T=3$ and $T=5$, respectively.

The first 2 weeks after inoculation are the crucial time to react to crop infection. Therefore, the $F_1$ scores elaborated for the first two weeks after inoculation (DPI from 1 to 14) were analyzed in the next step. The trend models (linear and quadratic) showing the increase in the effectiveness of the classification in the consecutive days after inoculation were adjusted to the obtained classification results. The examples of the $F_1$ scores obtained using the best-performing Ridge classifier for all analyzed variants and the aggregation interval of $T=3$ or 5 are presented in Fig. 7a).

The component diagrams in Fig. 7 reflect the results for the individual model variant (*CONTROL* vs each disease separately). Slightly better results of the fit were obtained for the aggregation period $T=5$ (coefficient of determination $R^2$ in the range 0.3 to 0.83, average 0.72), in comparison to the aggregation period $T=3$ (coefficient of determination $R^2$ in the range 0.2–0.72, average 0.56). The attempts were made to fit other trend lines to the analyzed results of $F_1$ scores in days post-inoculation, but—in the remaining cases—no satisfactory results were obtained.

Table 4 summarizes the results for the linear trends. The last column shows the point of intersection of the trend line with our acceptance threshold ($F_1$ score = 0.8). The point of intersection is the moment at which it can be assumed that a given crop is infected with the analyzed pathogens. If the point of the intersection has reached a value lower than the data aggregation range, the earliest possible moment of diagnosis of the infection was given in parentheses, which resulted from the interval of collecting the necessary measurement data for a specific case.

**Table 4:** Summary of the results of the analysis of the time of diagnosis of infection with the investigated pathogens; the earliest possible moment of diagnosis of the infection was given in parentheses.

Period	Disease	${{\textbf {R}}}^{2}$	Point of intersection for ${{\textbf {F}}}_{1}$ score = 0.8
3 days	*ALL*	0.72	6.2
5 days	*ALL*	0.30	3.4 (5)
3 days	Anthracnose	0.28	– (3)
5 days	Anthracnose	0.69	0.7 (5)
3 days	Bacterial speck	0.65	7.4
5 days	Bacterial speck	0.75	7.6
3 days	Early blight	0.20	– (3)
5 days	Early blight	0.56	– (5)
3 days	Late blight	0.55	2.8 (3)
5 days	Late blight	0.80	6.4
3 days	Septoria leaf spot	0.56	4.0
5 days	Septoria leaf spot	0.83	4 (5)

In some cases, the $F_1$ score of 0.8 was not reached by the trend model. The remaining results are promising and indicate the possibility of diagnosing the infection of the investigated pathogens in less than the accepted 7 days. The best results for the *ALL* vs *CONTROL* case were obtained for the data aggregation interval of $T=5$ (linear model: 5 days, quadratic model: 5.4 days). In the analysis of individual diseases, the best results were obtained for the following cases:

- Early blight—3 days ($T=3$),
- Late blight—3 days ($T=3$),
- Anthracnose—3 days ($$T=3$$).

For the remaining diseases, the best results were obtained for the following models and aggregation intervals:

- Septoria leaf sport—4 days (T = 3),
- Bacterial speck—8 days (T = 3).

## Discussion

In this study, the daily measurement frequency was applied while capturing the data, as it was found more reliable than the sporadic acquisition^6,7,12,14^ or weekly^5^ measurements. For the presented strategy of aggregating the datasets from consecutive measurement days, the decrease in the variance of the obtained results was noticeable ($T=3$ and $T=5$, the variance of 0.009 and 0.012, respectively). Increasing the frequency of the measurements, e.g., every 12–24 h as in the study by Gold et al.^10^, will likely have an even more significant impact on increasing the effectiveness of the constructed classification models. The obtained results confirm the possibility of early disease classification using hyperspectral data. The results were consistent with the previous studies—in the work by Nagasubramanian et al.^15^, the classification rate of 90% for $DPI=3$ was achieved. However, in this case, the recognition was made on stems, not leaves. This, in turn, resulted in the inability to use the discussed solution in remote sensing techniques because leaves are covering the stems. On the other hand, in the study reported by Xie et al.^4^, a classification rate of 90% was achieved for DPI = 4. However, it should be pointed out that in $DPI=5$, the classification score dropped to 33.33%. Finally, Gold et al.^10^, achieved very high accuracy (91.3%) for the group of 64 measurements defined as “Pre-symptomatic”.

The measurements used during the experiments were acquired in laboratory conditions (phytotrons), similar to other researches facing disease classification issue^4,6,9,10^. It is advisable to validate the machine learning models for crops under real conditions^6,8^. However, this task is much more challenging due to the limited possibilities of maintaining a controlled research environment and the elimination of various disturbances in crops related to the variability of conditions and the presence of additional factors (e.g., other pathogens). Additionally, the hyperspectral measurements were performed manually. This process was laborious and time-consuming, and—at the same time—it was subject to human error. Also, performing the calibration was necessary before each measurement series. Developing non-invasive remote sensing methods for diagnosing plant diseases requires the automation and standardization of such measurements. To improve the measurement process, unmanned aerial vehicles or walking robots could be utilized. Finally, using the recent deep learning advances could help better enhance the classification results (however, much more training data would be needed). Also, it would be interesting to understand which reflectance bands contribute to the classification process, hence should be considered important for specific diseases.

## Conclusions and future work

In this paper, the problem of non-invasive early detection and differentiation of *S. lycopersicum* diseases from hyperspectral data was tackled, which is crucial to decrease the negative effects of plant diseases, ultimately leading to yield reduction. The machine learning approaches to accelerate the process of detecting crop infection by appropriate disease recognition before the appearance of the symptoms becomes visible to the naked eye were introduced. The experiments revealed that the best-quality classification was obtained using the Ridge classifier—in the *ALL* vs *CONTROL* scenario, the $F_1$ score was 0.87 for $T=1$, 0.9 for $T=3$ and 0.88 for $T=5$. In addition, a significant improvement in the results and a reduction in variance using the proposed data aggregation method were noticed (for $T=3$ and $T=5$ days) compared to the approach using samples from a single measurement day only ($T=1$ day). For the period of the first two weeks from inoculation ($DPI=1...14$), the classification results improved significantly—the $F_1$ score for the classification of all diseases and control samples (*ALL* vs *CONTROL*) elaborated using the Ridge classifier and aggregation period T=3 amounted to:

- 0.71–0.95 (0.84 average) for $$DPI=[1,14]$$,
- 0.70–1.00 (0.92 average) for $$DPI=[15,63]$$.

Similar observations can be inferred for the separate classification of individual diseases. For $DPI=[1,14]$, the best mean result was obtained for *EB* (0.91), whereas for *AN*, *LB* and *SL* the results were 0.90, 0.89 and 0.86, respectively, and the worst results were elaborated for *BS* (0.82). For DPI = [15,63], the best mean result was obtained for *AN* (0.93), for *LB*, *SL* and *BS* the results were 0.92, 0.91 and 0.89, and the worst score for *EB*: 0.88. Overall, without divisions due to DPI, the best average results were obtained for *AN* (0.92). The worse outcomes were observed for *LB*, *SL* and *EB* (0.91, 0.90 and 0.89, respectively), and the worst for *BS* (0.86).

The results reported in this work constitute an interesting point of departure for further research, e.g., for other crops. It would be interesting to repeat the study with a greater frequency of measurements and with an increased sample size (this could be also achieved using various data augmentation routines^27^), and over other independent test sets to robustify the conclusions concerning the generalization capabilities of the developed machine learning models. Additionally, exploitation of deep learning techniques for this task is part of the future work^28^, considering that using such methods may significantly improve the classification performance. Also, the future efforts could focus on additional feature extraction and selection techniques, to better understand which part of the spectrum is pivotal in distinguishing specific diseases^29^. Finally, the enormous scalability of machine learning solutions can be achieved with on-board processing—the current research efforts are focused on developing the classification engines which will be deployed on board the Intuition-1 satellite, in a hardware-constrained execution environment^30^.

## Data Availability

The hyperspectral measurements presented in this study are available at https://bit.ly/3W7VroF.

## References

[1] Datar V, Mayee C (1982). Conidial dispersal of *Alternaria solani* in tomato. Indian Phytopathol..

[2] Grigolli JFJ (2011). Characterization of tomato accessions for resistance to early blight. Crop Breed. Appl. Biotechnol..

[3] Sharma, P. & Sharma, S. Paradigm shift in plant disease diagnostics: A journey from conventional diagnostics to nano-diagnostics. in *Part of the Fungal Biology book series (FUNGBIO)*, 237–264. 10.1007/978-3-319-27312-9_11 (Springer International Publishing, 2016).

[4] Xie C, Yang C, He Y (2017). Hyperspectral imaging for classification of healthy and gray mold diseased tomato leaves with different infection severities. Comput. Electron. Agric..

[5] Xie Y, Plett D, Liu H (2021). The promise of hyperspectral imaging for the early detection of crown rot in wheat. AgriEngineering.

[6] Bauriegel E, Giebel A, Geyer M, Schmidt U, Herppich W (2011). Early detection of Fusarium infection in wheat using hyper-spectral imaging. Comput. Electron. Agric..

[7] Jones C, Jones J, Lee W (2010). Diagnosis of bacterial spot of tomato using spectral signatures. Comput. Electron. Agric..

[8] Lu J, Ehsani R, Shi Y, de Castro AI, Wang S (2018). Detection of multi-tomato leaf diseases (late blight, target and bacterial spots) in different stages by using a spectral-based sensor. Sci. Rep..

[9] Ashourloo D, Matkan AA, Huete A, Aghighi H, Mobasheri MR (2016). Developing an index for detection and identification of disease stages. IEEE Geosci. Remote Sens. Lett..

[10] Gold KM (2020). Hyperspectral measurements enable pre-symptomatic detection and differentiation of contrasting physiological effects of late blight and early blight in potato. Remote Sens..

[11] Ashourloo D, Mobasheri MR, Huete A (2014). Evaluating the effect of different wheat rust disease symptoms on vegetation indices using hyperspectral measurements. Remote Sens..

[12] Peng Y (2022). Early detection of plant virus infection using multispectral imaging and spatial-spectral machine learning. Sci. Rep..

[13] Nguyen C (2021). Early detection of plant viral disease using hyperspectral imaging and deep learning. Sensors (Switzerland).

[14] Cen Y, Huang Y, Hu S, Zhang L, Zhang J (2022). Early detection of bacterial wilt in tomato with portable hyperspectral spectrometer. Remote Sens..

[15] Nagasubramanian K (2018). Hyperspectral band selection using genetic algorithm and support vector machines for early identification of charcoal rot disease in soybean stems. Plant Methods..

[16] Kuska M (2015). Hyperspectral phenotyping on the microscopic scale: Towards automated characterization of plant–pathogen interactions. Plant Methods..

[17] Smykała K, Ruszczak B, Dziubański K (2020). Application of ensemble learning to detect *Alternaria solani* infection on tomatoes cultivated under foil Tunnels. Intell. Environ..

[18] Ruszczak B, Smykała K, Dziubański K (2020). The detection of *Alternaria solani* infection on tomatoes using ensemble learning. J. Ambient Intell. Smart Environ..

[19] Wickham H (2014). Tidy data. J. Stat. Softw..

[20] Georgiev GT, Butler JJ (2007). Long-term calibration monitoring of Spectralon diffusers BRDF in the air-ultraviolet. Appl. Opt..

[21] Nawrocka, B., Robak, J., Ślusarski, C. & Macias, W. *Choroby i szkodniki pomidora w polu i pod osłonami* (Wydawnictwo Plantpress Sp. z o.o, Kraków, 2001).

[22] Zhu H (2017). Hyperspectral imaging for presymptomatic detection of tobacco disease with successive projections algorithm and machine-learning classifiers. Sci. Rep..

[23] Xu C, Ding J, Qiao Y, Zhang L (2022). Tomato disease and pest diagnosis method based on the Stacking of prescription data. Comput. Electron. Agric..

[24] Ruszczak B, Boguszewska-Mańkowska D (2022). Soil moisture a posteriori measurements enhancement using ensemble learning. Sensors.

[25] Kang J, Jin R, Li X, Zhang Y, Zhu Z (2018). Spatial upscaling of sparse soil moisture observations based on ridge regression. Remote Sensing.

[26] Allwright, S. What is a good F1 score and how do I interpret it? (2022).

[27] Nalepa J, Myller M, Kawulok M (2020). Training- and test-time data augmentation for hyperspectral image segmentation. IEEE Geosci. Remote Sens. Lett..

[28] Tulczyjew L, Kawulok M, Longepe N, Saux BL, Nalepa J (2022). Graph neural networks extract high-resolution cultivated land maps from sentinel-2 image series. IEEE Geosci. Remote Sens. Lett..

[29] Ribalta Lorenzo P, Tulczyjew L, Marcinkiewicz M, Nalepa J (2020). Hyperspectral band selection using attention-based convolutional neural networks. IEEE Access..

[30] Nalepa J (2021). Towards on-board hyperspectral satellite image segmentation: Understanding robustness of deep learning through simulating acquisition conditions. Remote Sensing..

